# Atomic Layer Deposition Coating of Carbon Nanotubes with Aluminum Oxide Alters Pro-Fibrogenic Cytokine Expression by Human Mononuclear Phagocytes *In Vitro* and Reduces Lung Fibrosis in Mice *In Vivo*

**DOI:** 10.1371/journal.pone.0106870

**Published:** 2014-09-12

**Authors:** Alexia J. Taylor, Christina D. McClure, Kelly A. Shipkowski, Elizabeth A. Thompson, Salik Hussain, Stavros Garantziotis, Gregory N. Parsons, James C. Bonner

**Affiliations:** 1 Environmental and Molecular Toxicology Program, Department of Biological Sciences, North Carolina State University, Raleigh, North Carolina, United States of America; 2 Department of Chemical & Biomolecular Engineering, North Carolina State University, Raleigh, North Carolina, United States of America; 3 Clinical Research Unit, National Institute of Environmental Health Sciences, Research Triangle Park, North Carolina, United States of America; Duke University Medical Center, United States of America

## Abstract

**Background:**

Multi-walled carbon nanotubes (MWCNTs) pose a possible human health risk for lung disease as a result of inhalation exposure. Mice exposed to MWCNTs develop pulmonary fibrosis. Lung macrophages engulf MWCNTs and produce pro-fibrogenic cytokines including interleukin (IL)-1β, IL-6, tumor necrosis factor (TNF)-α, and osteopontin (OPN). Atomic layer deposition (ALD) is a novel process used to enhance functional properties of MWCNTs, yet the consequence of ALD-modified MWCNTs on macrophage biology and fibrosis is unknown.

**Methods:**

The purpose of this study was to determine whether ALD coating with aluminum oxide (Al2O3) would alter the fibrogenic response to MWCNTs and whether cytokine expression in human macrophage/monocytes exposed to MWCNTs in vitro would predict the severity of lung fibrosis in mice. Uncoated (U)-MWCNTs or ALD-coated (A)-MWCNTs were incubated with THP-1 macrophages or human peripheral blood mononuclear cells (PBMC) and cell supernatants assayed for cytokines by ELISA. C57BL6 mice were exposed to a single dose of A- or U-MWCNTs by oropharyngeal aspiration (4 mg/kg) followed by evaluation of histopathology, lung inflammatory cell counts, and cytokine levels at day 1 and 28 post-exposure.

**Results:**

ALD coating of MWCNTs with Al2O3 enhanced IL-1β secretion by THP-1 and PBMC in vitro, yet reduced protein levels of IL-6, TNF-α, and OPN production by THP-1 cells. Moreover, Al2O3 nanoparticles, but not carbon black NPs, increased IL-1β but decreased OPN and IL-6 in THP-1 and PBMC. Mice exposed to U-MWCNT had increased levels of all four cytokines assayed and developed pulmonary fibrosis by 28 days, whereas ALD-coating significantly reduced fibrosis and cytokine levels at the mRNA or protein level.

**Conclusion:**

These findings indicate that ALD thin film coating of MWCNTs with Al2O3 reduces fibrosis in mice and that in vitro phagocyte expression of IL-6, TNF-α, and OPN, but not IL-1β, predict MWCNT-induced fibrosis in the lungs of mice in vivo.

## Introduction

Multi-walled carbon nanotubes (MWCNTs) are fiber-like, engineered graphene nanomaterials that have a wide range of applications in engineering, electronics, and medicine. They are currently being utilized for their superior mechanical strength, large surface area and electrical conducting properties in many consumer products and for industrial purposes [1]. MWCNTs also have potential for biomedical applications, including drug delivery and scaffolds for tissue regeneration [2], [3]. Human exposure to MWCNTs will be inevitable due to increased production and use in a variety of consumer products, so it is extremely important to better understand the potential risks of MWCNTs to human health in order to ensure safe design of materials containing MWCNTs [4].

MWCNTs can be modified or ‘functionalized’ in a variety of ways to enhance mechanical and electronic properties, or drug delivery and imaging capabilities [5], [6]. Atomic layer deposition (ALD) thin-film coating with oxides, metals, and hybrid metal/organic materials is a method to modify MWCNTs to enhance conductivity, photovoltaic or catalytic applications, and attachment of biomolecules [7]–[9]. For example, aluminum oxide (Al_2_O_3_) and titanium oxide change surface functionality and wetting properties of organic fibers and allows for attachment with biomolecules by making them more hydrophilic [7], [8]. Zinc oxide or titanium oxide coating gives MWCNTs increased photosensitivity for photovoltaic or catalytic applications [8]. “Hybrid” organic/inorganic thin film coatings are also being explored and may enhance properties of MWCNTs. ALD was initially developed for use in the semiconductor industry [10], [11]. This process for depositing a thin-film is desirable for work at the nano-scale because it results in a highly uniform coating via a sequence of self-limiting reactions [7], [11]. Coating thickness increases with the number of ALD cycles. ALD coating of MWCNTs has also recently been used in microelectronics, enhancing conductivity, attachment of biomolecules and energy storage applications [12]. Due to the variety of applications that ALD thin-film coating provides, it is important to determine potential cytotoxic effects in a biological system.

MWCNTs have yet to be linked to human disease, but increasing evidence from *in vivo* rodent studies indicate that these engineered nanomaterials cause lung inflammation and fibrosis [13]–[19]. The majority of MWCNTs delivered to the lungs of mice by inhalation are engulfed by lung macrophages, which migrate to the distal alveolar regions of the lung and also translocate to the subpleural regions at the lung periphery to mediate subpleural fibrosis and inflammation [15]. MWCNTs can persist in the lungs of rodents for months after exposure to cause progressive inflammation and fibrosis [16], [17], [18]. This is likely due to impaired macrophage clearance of MWCNTs from the lung. Long MWCNTs cause ‘frustrated’ phagocytosis when engulfed by macrophage and length is likely an important factor in determining the pathogenicity of MWCNTs [20].

Lung macrophages produce a variety of cytokines that play important roles in inflammation, tissue repair, or the pathogenesis of lung fibrosis [21]. MWCNTs have been reported to stimulate the production of pro-inflammatory or pro-fibrogenic cytokines by macrophages, including interleukin (IL)-1β, IL-6, tumor necrosis factor (TNF)-α, and osteopontin (OPN) [21]–[24]. While macrophages are the primary clearance mechanism for removing MWCNTs and provide an important innate immune response in the lungs, they may also mediate disease pathogenesis through the production of pro-inflammatory and pro-fibrogenic cytokines [21].

No previous studies have been performed to determine how ALD layer coating affects the toxicity of MWCNTs and their potential to cause lung fibrosis. The purpose of this study was to determine whether ALD coating of MWCNTs with Al_2_O_3_ alters the expression of pro-inflammatory and pro-fibrogenic cytokines by human THP-1 mononuclear cells or human primary peripheral blood monocytes (PBMCs) *in vitro* and whether ALD coating alters the pro-fibrogenic potential of MWCNTs in the lungs of C57BL6 mice *in vivo*. We report that ALD coating of MWCNTs with Al_2_O_3_ enhanced IL-1β production by THP-1 cells and PBMCs, but decreased IL-6, TNF-α, and OPN expression. Moreover, ALD-coating reduced MWCNT-induced expression of all four cytokines in the lungs of mice and ALD-coated MWCNTs caused significantly less fibrosis in the lungs of mice. These results suggest that ALD thin film coating of with Al_2_O_3_ would reduce the pro-fibrogenic hazard of MWCNTs to humans.

## Methods

### Nanomaterials

Multi-walled carbon nanotubes (MWCNT) 0.5–40 µm in length synthesized by chemical vapor deposition were purchased from Helix Materials Solutions (Richardson, TX). Characterization of the MWCNTs was provided by the manufacturer and verified by Millennium Research Laboratories (Woburn, MA) and is described in our previously work [14]–[16]. Aluminum oxide nanoparticles (∼60 nm) were purchased from Sun Innovations (Fremont, CA). The Carbon black nanoparticles (CBNP), ∼8 nm, were from Columbian Chemicals as Raven 5000 Ultra II Powder (Marietta, GA) and have been described previously [14]–[15]. MWCNTs were surface coated with aluminum oxide via atomic layer deposition (ALD) [9]. The aluminum oxide layer is achieved using sequential saturation exposures of trimethylaluminum (Al(CH_3_)_3_) and water, separated by inert gas purging steps. The details of ALD coating of carbon nanotubes have been previously described [25].

### Preparation of MWCNTs and Nanoparticles

Uncoated & coated MWCNTs, carbon black nanoparticles, and aluminum oxide nanoparticles were weighed using a milligram scale (Mettler, Toledo OH) suspended in a sterile, 0.1% pluronic F-68 (Sigma-Aldrich, St. Louis MO) in phosphate buffer solution to achieve the final concentration of 10 mg/ml. Vials containing the suspended nanomaterials were either used as is (unsonicated) or dispersed using a cuphorn sonicator (Qsonica, Newton CT) at room temperature for 1 minute prior to dosing. A limulus amebocyte lysate (LAL) chromogenic assay (Lonza Inc., Walkersville MD) was used to test the nanomaterials for endotoxin contamination as we have previously validated [15] All MWCNTs, both uncoated and ALD-coated, as well as Al_2_O_3_ and CB nanoparticles, tested negative (<0.3 EU/ml) for endotoxin.

### Characterization of MWCNT Aggregate Size and Zeta Potential in Aqueous Media

MWCNTs were suspended in dispersion medium (0.1% pluronic/RPMI 1640 medium) as described for THP-1 exposures. Dynamic Light Scattering (DLS) and Zeta potential analyses were performed using ZetaSizer Nano (Malvern Instruments, Westborough, MA, USA) as described by us previously [26] (**Table S1**).

### Cell Culture and Nanoparticle Treatment

#### Human THP-1 Cells

THP-1 cells were purchased from ATCC (Manassas, VA) and cultured according to a standard protocol used by the NIEHS Nano GO Consortium [27]. Cells were cultured in suspension with RPMI-1640 Medium (Invitrogen, Carlsbad CA) containing 10% fetal bovine serum (Gibco) at 37°C and 5% CO_2_. Once confluent, the THP-1 cells were differentiated into a macrophage-like cell using 150 nM of 1α, 25-Dihydroxy-Vitamin D_3_ (EMD Millipore, Billerica MA). After the cells become semi-adherent, 10 ng/ml of lipopolysaccharide (LPS) (Sigma-Aldrich, St. Louis MO) was added to induce intracellular pro-IL-1β as well as 5 nM of phorbol 12-myristate 12-acetate (PMA) (Sigma-Aldrich) to further differentiate the monocytes to macrophages. Prior to nanoparticle exposure, 1×10^5^ cells were seeded into 96-well plates (Costar, Corning NY). THP-1 cells were then exposed to increasing doses (5, 10, 50 & 100 µg/ml) of uncoated and Al_2_O_3_ coated MWCNTs (unsonicated vs. sonicated) as well as carbon black & Al_2_O_3_ nanoparticles (negative & positive control) for 24 hours. Cells were given a dose that was determined by the estimated number of nanotubes. Cell culture conditioned medium were collected at 24 hr for ELISA. MTS cytotoxicity assays were performed on the monolayer of cells (**Figs S1-S3**).

#### Primary Human Monocytes

Adult human donors without any history of chronic illness or current use of medication were recruited to the Clinical Research Unit of the National Institute of Environmental Health Sciences (NIEHS). The protocol was approved by the Institutional Review Board of the NIEHS. Primary human monocytes from the blood of human donors were isolated following the protocol described by us previously [26]. Briefly, mononuclear cells were isolated using Histopaque (Sigma Aldrich) and CD14^+^ monocytes were separated using CD14 magnetic beads (Miltenyi Biotec, Boston MA). Monocyte viability and purity were confirmed using flow cytometry and cytospin preparations (95–99% viable, 92–95% pure). CD14^+^ cells were seeded at a density of 500,000 cells per well in 24-well culture plates in *ex vivo* medium (Lonza) supplemented with 1% human serum and antibiotics (1% solution of penicillin (100 µg/mL) and streptomycin (100 µg/mL), Invitrogen). Cells were allowed to adhere to the plates in an incubator at 37°C, 5% CO_2_, and 95% relative humidity for 2 hrs. After 2 hrs, the cell culture medium was aspirated, cells were washed with fresh medium and incubated with 5 ng/mL of ultrapure LPS from *Escherichia coli* O111:B4 (List Biological Laboratories, Inc., Campbell, CA) over night for priming. After 24 h the cells were treated with uncoated MWCNTs, Al_2_O_3_-coated MWCNTs, CB NPs, or Al_2_O_3_ NPs.

### Exposure of Mice to MWCNTs and Processing of Lung Tissue

Mice (C57BL6, Jackson Laboratories) were exposed to uncoated MWCNTs or Al_2_O_3_-coated MWCNTs at 4 mg/kg in 0.1% Pluronic (Sigma-Aldrich) surfactant solution or 0.1% Pluronic alone via oropharyngeal aspiration while under isoflurane anesthesia. Mice were euthanized via intraperitoneal injection of Fatal Plus (Vortech Pharmaceuticals, Dearborn, MI) on day 1 or day 28 after MWCNT exposure. At necropsy, the lungs were lavaged with Dulbecco's Phosphate Buffered Saline (DPBS) and bronchoalveolar lavage fluid (BALF) was collected for cell differential counts and enzyme-linked immunosorbent assay (ELISA). The middle and caudal lobes of the right lung were collected for mRNA analysis and stored in RNAlater as per manufacturer's instructions (Ambion, Austin, TX). The left lungs were intratracheally infused with 10% neutral buffered formalin and fixed for 24 hrs and then transferred to 70% ethanol. The left lung was cross-sectionally cut into three portions, embedded in paraffin and sectioned and stained with either hematoxylin and eosin (H&E) or Masson's trichrome to visualize collagen deposition in the lungs.

### Bronchoalveolar Lavage and Cytology

Lungs were serially lavaged two times with 0.5 ml DPBS. The lavages were combined and a 0.1 ml sample was used for differential cell counts. A Thermo Scientific Cytospin 4 (ThermoFisher Scientific, Waltham, MA) was used to pellet cells from the BALF sample onto glass slides followed by fixation and staining with the Diff-Quik Stain Set (Dade Behring Inc, Newark, DE). Differential counting to identify relative percentages of macrophages, neutrophils, eosinophils and lymphocytes was performed on BALF cells fixed and stained on slides after cytospin centrifugation. 500 cells were counted for each BALF sample. The differential cell counts were then averaged for each dose group and represented as mean±SEM. The relative percentage of mouse lung macrophages containing MWCNTs was also evaluated by counting 100 cells per BALF sample fixed and stained on cytospin slides.

### Scoring of Fibrotic Lung Lesions

The scoring system for lung fibrosis at 28 days used in this study is an unbiased method that we have employed previously [16]. Three sections of lung, one each from the cranial, middle, and caudal portions of the left lung lobe, from each mouse were evaluated in a blinded fashion. The lungs were scored for the amount of collagen present (based on Masson's trichrome-stained sections), the thickness of the alveolar walls, and the number of fibroblast-like cells associated with the particle-associated lesions. These scores were averaged and the average scores were then divided by the relative scores for the amount of nanoparticles present in the lungs to give an adjusted average score for each animal. All scores were relative scores on a scale from 0–4, with zero representing the levels of these parameters in the PBS control group, 1 representing minimal change, 2 representing mild change, 3 representing moderate change, and 4 representing marked change.

### Determination of Cell Viability

The CellTiter 96 AQ_ueous_ One Solution Cell Proliferation Assay (MTS assay, Promega, Madison WI) was used (according to the manufacturer's protocol) to measure THP-1 cell and PBMC survival following exposure to the nanotubes and nanoparticles. To avoid nanoparticle interference with absorbance measurements, supernatants were centrifuged (15000xg for 5 min) and transferred to a new 96-well plate. Formazan absorbance was measured at 450 nm on the Multiskan EX microplate spectrophotometer (ThermoFisher Scientific, Waltham, MA). Values were expressed as mean ± SEM.

### Transmission Electron Microscopy

THP-1 cells exposed to uncoated and coated MWCNTs for 24 hr were post-fixed in 1% osmium tetroxide in 0.1 M sodium phosphate buffer, pH 7.2, dehydrated through graded ethanol solutions, cleared in acetone, and then infiltrated and embedded in Spurr's resin. Unstained thin sections were mounted on copper grids and then examined using a Philips EM208S transmission electron microscope.

### ELISA

IL-1β, IL-6, OPN and TNF-α protein levels in cell culture conditioned medium were measured by ELISA (R&D Systems, Minneapolis, MN). A 24 hr time point was chosen based on preliminary experiments that determined the optimal time for cytokine production by THP-1 cells exposed to uncoated and ALD-coated MWCNTs over a time course (4, 24, 48 hrs). Samples were diluted and assayed according to the kit instructions. Absorbance was read at 450 nm by the Multiskan EX microplate spectrophotometer (ThermoFisher Scientific) with a correction wavelength set at 540 nm. Linear regression analysis was performed to interpolate value (in pg/ml) from the standard curve.

### Data and Statistical Analysis

Data was converted into graphs and statistical analysis was performed using GraphPad Prism software version 5.00 (GraphPad Software Inc., San Diego CA). A one-way ANOVA with a *post hoc* Tukey was used to identify significant differences between the controls and treatments. A two-way ANOVA with a post Bonferroni test were used to identify significant differences among treatment groups. The significance was set at *p*<0.05 unless stated otherwise. Values were expressed as mean ± SEM.

### Ethics Statement

The protocol for collection of human peripheral blood monocytes was approved by the Institutional Review Board of the National Institute of Environmental Health Sciences (NIEHS). Participants provided their written informed consent to participate in this study. The original signed consent forms are stored at the National Institutes of Health Clinical Center (NIH CC) in Bethesda, MD, and a scanned version is stored in the NIH Clinical Research Information Center (CRIS) electronic system. All studies using mice were approved by the North Carolina State University IACUC (protocol no. 13-086-B). No adverse health or behavior reactions were observed during the experiments with mice.

## Results

### Surface modification of MWCNTs via atomic layer deposition (ALD) results in uniform and stable nanoscale coating of aluminum oxide

**Figure 1:**
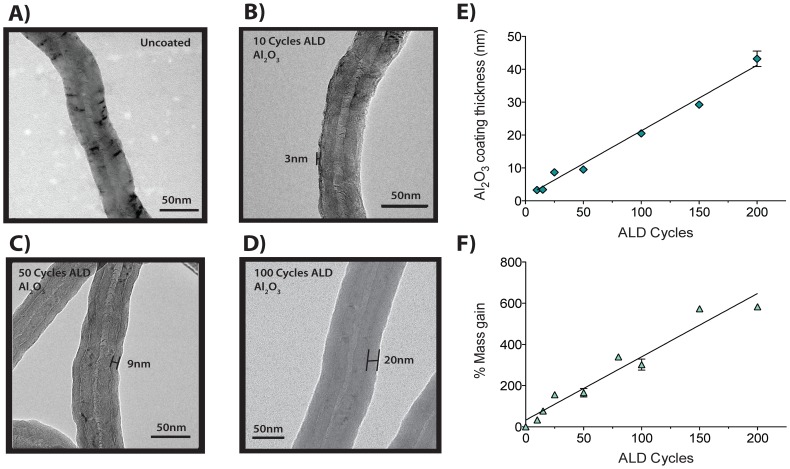
Atomic Layer Deposition Functionalization of MWCNTs. **A**) Transmission electron microscopy (TEM) image of MWCNTs from Helix that went through the ALD process with no coating added. **B**) TEM image of a semi-uniform ALD-coating of Al_2_O_3_ on MWCNT; 10 cycles of ALD resulted in a 3 nm thick coating. **C**) TEM image of a uniform ALD-coating of Al_2_O_3_ on MWCNT; 50 cycles of ALD resulted in a 9 nm thick coating. **D**) TEM image of a uniform ALD-coating of Al_2_O_3_ on MWCNT; 100 cycles of ALD resulted in a 20 nm thick coating. **E**) Al_2_O_3_ coating thickness (nm) on MWCNTs as a function of ALD cycle. Data represent mean values (±SEM) with r^2^ = 0.9618. **F**) Percent mass gain with increasing ALD cycles. Data represent mean values (±SEM) with r^2^ = 0.9471.

The ALD process involves sequential, alternating doses of trimethylaluminum (TMA) and water to provide a precise thickness control coating at the nanoscale [7], [9], [11]. Increasing cycles of ALD proportionally increased the thickness of the Al_2_O_3_ coating on MWCNTs, proportionally increased nanotube mass, and caused MWCNTs to break along the radial axis during sonication. Transmission electron microscopy (TEM) revealed the nanoscale precision of Al_2_O_3_ thin film coating achieved on MWCNTs after increasing number of ALD cycles (**Fig. 1B-D**). Uncoated (U)-MWCNTs were used as the control to compare to the Al_2_O_3_ coated (A)-MWCNTs (**Fig. 1A**) and went through the same ALD process as the A-MWCNTs, but without TMA added to the reaction. TEM imaging shows that 10 ALD cycles results in a coating with an average thickness of 3 nm (**Fig. 1B**). 50 ALD cycles results in a thicker coating, approximately 9 nm in diameter (**Fig. 1C**). The thickest coating (∼20 nm) of the A-MWCNT used in the current experiments was achieved with 100 ALD cycles (**Fig. 1D**). Quantitative measurements showed that increasing ALD cycles resulted in a corresponding cycle-dependent increase in Al_2_O_3_ coating thickness (**Fig. 1E**) as well as an increase in mass (**Fig. 1F**). A-MWCNTs suspended in aqueous medium (RPMI) had an increased average hydrodynamic diameter, but decreased zeta potential, compared to U-MWCNTs (**Table S1**). MWCNTs tested negative for endotoxin contamination as determined by ameobocyte lysate test (Associates of Cape Cod, East Falmouth, MA), before and after ALD coating with Al_2_O_3_ (data not shown).

### Al_2_O_3_-coated MWCNTs and uncoated MWCNTs are taken up by THP-1 macrophages

**Figure 2:**
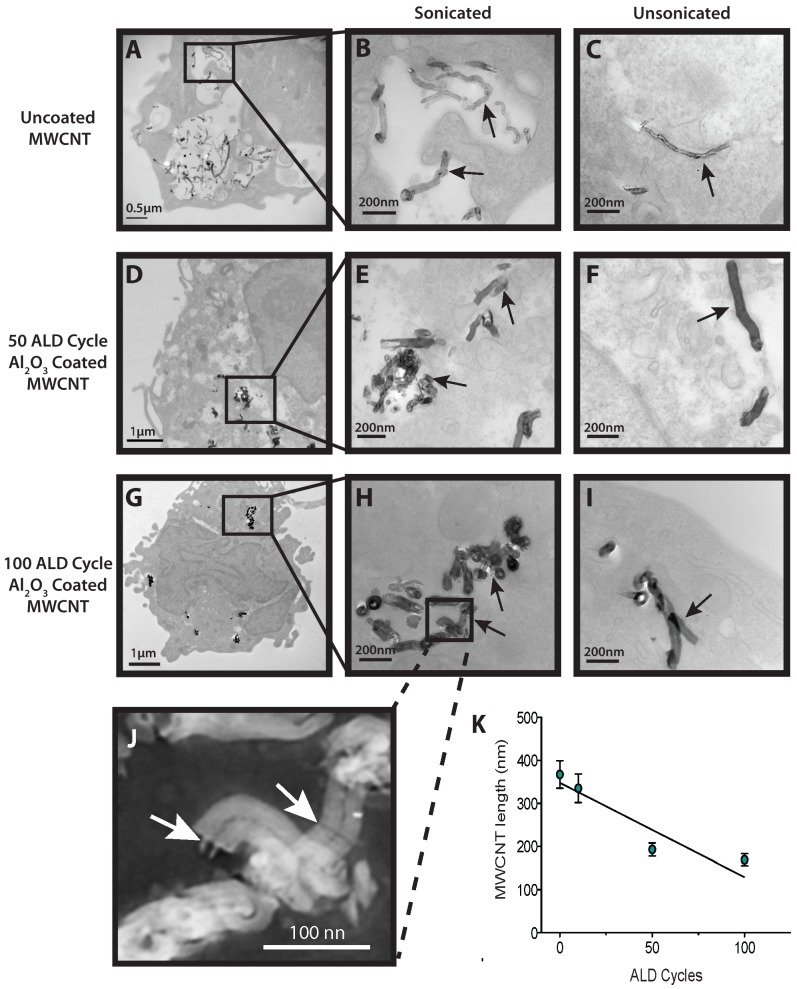
Transmission electron microscopy of human macrophages engulfing MWCNTs. **A**) Lower magnification (x18000) of uncoated, sonicated MWCNTs contained in vesicles within a THP-1 macrophage. **B**) Higher magnification (x56000) of uncoated, sonicated MWCNTs within a vesicle of a THP-1 macrophage. **C**) High magnification (x56000) of a single (unsonicated) MWCNT within the cytoplasm of a THP-1 macrophage. **D**) Lower magnification (x7100) of a THP-1 macrophage with sonicated Al_2_O_3_ coated MWCNTs (50 ALD cycles) in the cytoplasm. **E**) Higher magnification (x56000) of a cluster of sonicated Al_2_O_3_-coated MWCNT fragments (50 ALD cycles within the cytoplasm of a THP-1 macrophage. **F**) High magnification (x36000) of a THP-1 macrophage with Al_2_O_3_ coated MWCNTs (50 ALD cycles –unsonicated) in the cytoplasm in close proximity to the nucleus. **G**) Low magnification (x7100) of a macrophage with Al_2_O_3_-coated (100ALD cycles) sonicated MWCNTs in the cytoplasm. **H**) Inset showing a higher magnification (x56000) of a cluster of sonicated Al_2_O_3_-coated MWCNTs (100 ALD cycles) within the cytoplasm of a macrophage. **I**) High magnification (x56000) showing unsonicated Al_2_O_3_-coated (100 ALD cycles) MWCNTs in the cytoplasm of a THP-1 macrophage. **J**) High magnification inverted image of ALD-coated MWCNTs from panel H showing fracture points of breakage (arrows). **K**) Al_2_O_3_ coated MWCNT length (nm) as a function of ALD cycle post sonication. Data represent mean values (±SEM) of 20 measurements of nanotubes thickness and length.

Transmission electron microscopy of human macrophages demonstrated uptake of both the U- and A-MWCNTs 24 hrs post exposure (**Fig. 2A-I**). A lower magnification (x18000) photomicrograph shows that numerous U-MWCNTs dispersed by sonication were engulfed by THP-1 macrophages and contained within cytoplasmic vesicles (**Fig. 2A & B**). Unsonicated, U-MWCNTs were taken up as well, but fewer were observed within the macrophages (**Fig. 2C**). The THP-1 cells also engulfed 50 ALD cycle A-MWCNTs after 24 hrs (**Fig. 2D-F**). A-MWCNTs retained the Al_2_O_3_ coating after sonication and phagocytosis by THP-1 cells (**Fig. 2E, H**). Unsonicated, A-MWCNTs taken up by the THP-1 cells were approximately of the same length range as U-MWCNTs. Unsonicated A-MWCNTs coated with 50 ALD cycles were observed within the cytoplasm of THP-1 cells in close proximity to the nucleus (**Fig. 2F**). THP-1 cells also engulfed the sonicated 100 ALD cycle A-MWCNTs (**Fig. 2G**) and a higher magnification showed that the Al_2_O_3_ coating was still intact on the surface of the A-MWCNTs after being taken up by THP-1 cells (**Fig. 2H**). Very few unsonicated 100 ALD cycle A-MWCNTs were taken up by THP-1 cells (**Fig. 2I**). Interestingly, sonication of the A-MWCNTs resulted in breakage and a significantly reduced length of nanotubes coated with 50 or 100 ALD cycles (**Fig. 2J & K**). A-MWCNTs coated with 10 ALD cycle were also phagocytized by the macrophages (data not shown).

### Al_2_O_3_ coating alters the cytotoxic response of THP-1 cells to MWCNTs

The MTS assay was utilized to test the cytotoxic effect of the MWCNTs on THP-1 cells at 24 hrs post-exposure. All MWCNT treatments caused significant cytotoxicity (P<0.05 or greater compared to control media) at the highest doses (50 and 100 µg/ml) when determined by estimated nanoparticle number. Sonicated MWCNTs caused greater cytotoxicity as compared to unsonicated MWCNTs, especially when the MWCNT dose was determined by estimated nanoparticles number (**Fig. S1**). This decrease in cell viability after exposure to the 10 cycle A-MWCNTs was the most significant at the higher doses (50 and 100 µg/ml). The cytotoxic response of THP-1 cells to 50 ALD cycle A-MWCNTs was comparable to the U-MWCNTs when dose was determined by mass, but similar to the 10 ALD coating when the dose was based on estimated nanoparticle number. However, the 100 ALD cycle A-MWCNTs appeared to have little to no cytotoxic effect when the cells were given a dose determined by mass. When the dose was determined by nanoparticles number, the response was comparable to the 10 or 50 ALD cycle A-MWCNTs. In general, dispersal of the nanotubes via sonication caused a more cytotoxic effect when compared to unsonicated nanotubes. Without dispersal via sonication, nanotubes formed aggregates, which were not avidly engulfed by macrophages.

### Al_2_O_3_-coated MWCNTs increase IL-1β production but decrease IL-6, OPN, and TNF-α secretion by THP-1 cells *in vitro*

**Figure 3:**
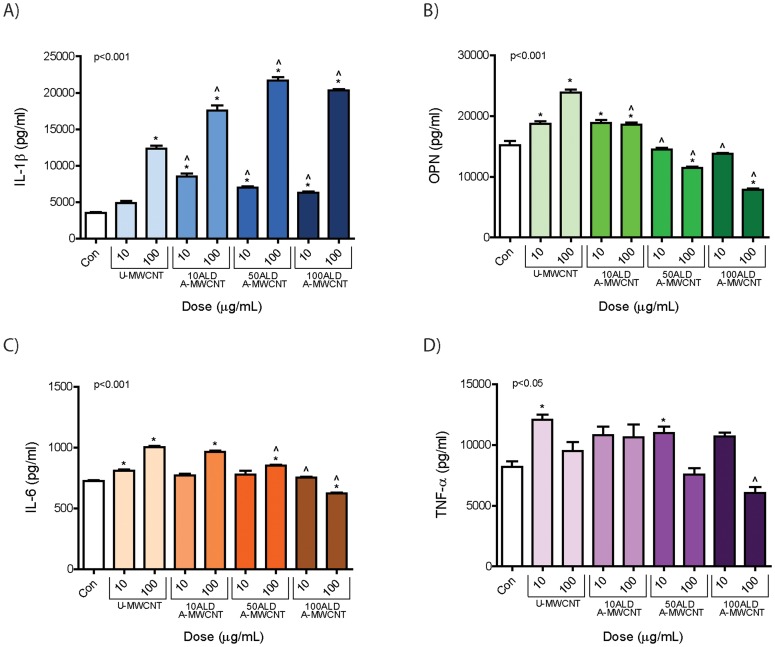
Cytokine protein levels secreted by THP-1 cells 24 hr after exposure to MWCNTs coated with Al_2_O_3_ by ALD. THP-1 cells were exposed to low (10 µg/ml) and high (100 µg/ml) doses of uncoated MWCNTs (U-MWCNT) or atomic layer deposition (ALD)-coated MWCNTs (A-MWCNT) functionalized with increasing layers of Al_2_O_3_ achieved by 10, 50, or 100 ALD cycles (A-MWCNT). ELISAs were performed on cell supernatants for **A**) IL-1β, **B**) OPN, **C**) IL-6 and **D**) TNF-α. Data are representative graphs of three separate experiments and are expressed as means ± SEM. P values are indicated for each graph. *Significant compared to control. ∧Significant effect between A-MWCNT and U-MWCNT at the same dose.

THP-1 cells were exposed to sonicated U-MWCNTs or A-MWCNTs at 10 and 100 µg/ml. Based on the linear mass gain to ALD coating cycle relationship shown in **Fig. 1**, the mass dose was corrected by estimating numbers of nanotubes per unit mass for each series of ALD coating cycles relative to U-MWCNTs to correct for the increase in mass. After 24 hr the cell supernatant was collected to evaluate secreted cytokine protein levels via ELISA. U-MWCNTs increased the secretion of IL-1β, IL-6, OPN, and TNF-α (**Fig. 3**). Dosing cells with A-MWCNTs increased IL-1β secretion by THP-1 cells as compared to U-MWCNTs, but decreased secretion of IL-6, OPN, and TNF-α.

### Al_2_O_3_ nanoparticles increases IL-1β production but decreases IL-6, OPN, and TNF-α secretion by THP-1 cells *in vitro*

**Figure 4:**
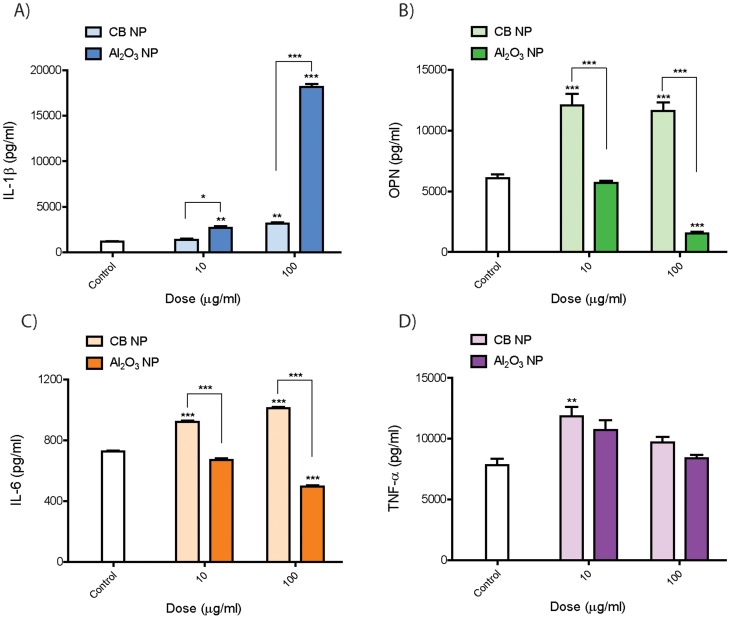
Cytokine protein levels secreted by THP-1 cells 24 hr after exposure to Al_2_O_3_ or carbon black nanoparticles (CB NP). THP-1 cells were exposed to low (10 µg/ml) and high (100 µg/ml) doses of Al_2_O_3_ or CB NP. ELISAs were performed on cell supernatants for ELISAs were performed on cell supernatants for **A)** IL-1β, **B)** OPN, **C)** IL-6 and **D)** TNF-α. Data are representative graphs of three separate experiments and are expressed as means ± SEM. Asterisk directly above bar indicate comparison to control, whereas asterisk over connecting bar indicate comparison between CB NP and Al_2_O_3_ NP. *P<0.05, **P<0.01, ***P<0.001. Data are representative of three experiments and expressed as means ± SEM.

Carbon black and Al_2_O_3_ nanoparticles both resulted in a moderate cytotoxic response after 24 hrs (**Fig. S2**). Al_2_O_3_ nanoparticles also caused a robust and highly significant dose-dependent increase in IL-1β secretion by macrophages (**Fig. 4**). Carbon black increased IL-1β in a dose-dependent manner but only slightly when compared to Al_2_O_3_ nanoparticles. However, carbon black nanoparticle exposure resulted in a significant increase in IL-6, OPN, and TNF-α. In contrast, Al_2_O_3_ nanoparticles caused a significant decrease in IL-6 and OPN levels but did not significantly alter TNF-α secretion by THP-1 cells.

### Al_2_O_3_-coated MWCNTs increase IL-1β but reduce OPN secretion by human peripheral blood monocytes

**Figure 5:**
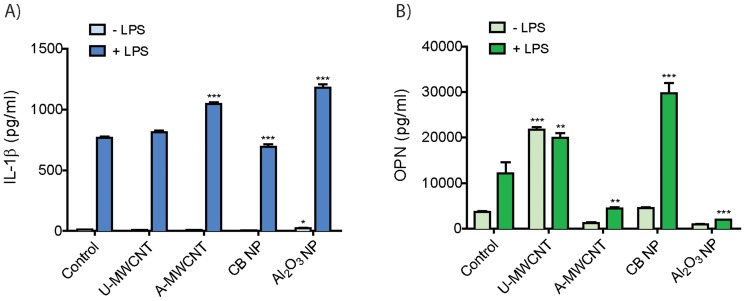
Effect of Al_2_O_3_-coated MWCNTs or Al_2_O_3_ nanoparticles on the secretion of cytokines by primary human blood monocytes (PBMCs). Human PBMCs were treated with or without LPS for 2 hrs then exposed to nanoparticles for 24 hrs prior to collecting cell supernatants for ELISA. A single nanoparticle dose of 100 µg/ml was used and corrected for estimated particle number to account for the difference in mass between uncoated ‘U-MWCNT’ and 10 ALD cycle Al_2_O_3_-coated ‘A-MWCNT’. ELISAs were performed on cell supernatants for **A)** IL-1β and **B)** OPN. Data are expressed as the mean +/− SEM of quadruplicate cultures of PBMCs. *P<0.05, **P<0.01, ***P<0.001 compared to corresponding controls (−LPS or +LPS).

Primary CD14^+^ human peripheral blood monocytes (PBMCs) were collected from normal human donors and cultured *in vitro* prior to priming with 5 ng/ml LPS as described in *Methods*. For this experiment we used a single dose of nanoparticles (100 µg/ml). A-MWCNTs were coated with 10 ALD cycles of Al_2_O_3_ and corrected for estimated nanoparticle number relative to U-MWCNT. A-MWCNTs or Al_2_O_3_ nanoparticles, but not U-MWCNTs, produced a significant increase in IL-1β secretion by LPS-primed PBMCs (**Fig. 5A**). OPN levels secreted by human PBMCs were increased by LPS priming with exposure to either U-MWCNTs or CB nanoparticles. In contrast, A-MWCNTs or Al_2_O_3_ nanoparticles markedly suppressed OPN secreted by PBMCs (**Fig. 5B**). While the IL-1β and OPN responses of PBMCs were similar to THP-1 cells, the PBMCs did not show significant differences in IL-6 or TNF-α secretion between U- and A-MWCNTs (data not shown). The MTS assays showed that U-MWCNTs, A-MWCNTs, and Al_2_O_3_ nanoparticles caused less than 20% cytotoxicity in PBMCs, while CB nanoparticles caused as much as 50% cytotoxicity in PBMCs (**Fig. S3**).

### Al_2_O_3_ coating decreases the pro-fibrogenic effect of MWCNTs but does not alter acute inflammation *in vivo*

**Figure 6:**
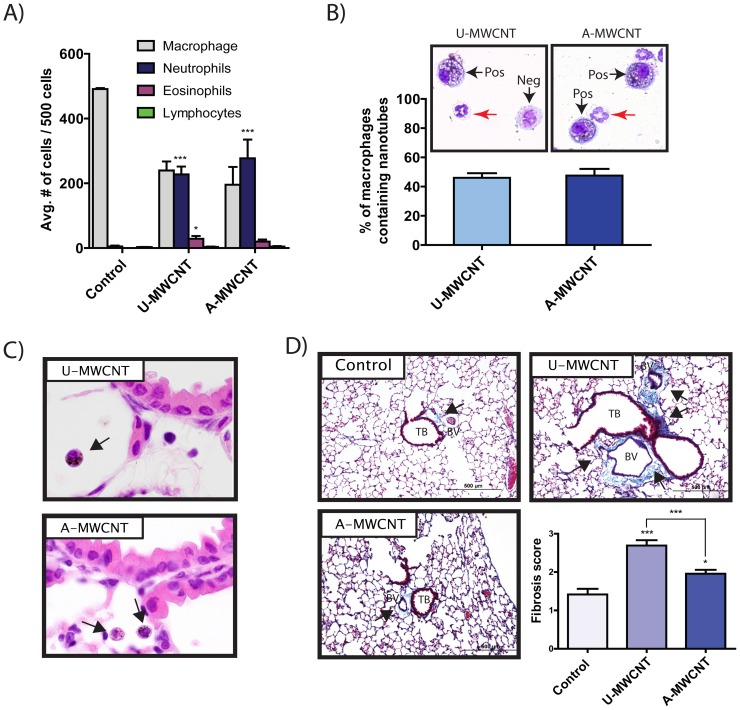
Effect of Al_2_O_3_-coated MWCNTs on Lung Inflammation and Fibrosis in C57BL6 mice. Uncoated (U)-MWCNT or MWCNT coated with Al_2_O_3_ by 50 ALD cycles (A-MWCNT) were delivered to the lungs of mice by oropharyngeal aspiration (4 mg/kg) and necropsy performed at 1 or 28 days post-exposure to collect bronchoalveolar lavage fluid (BALF) and lung tissues for histopathology. **A**) Inflammatory cell differential counts in 1 day BALF demonstrated that U-MWCNTs caused primarily a neutrophilic inflammatory response that was not significantly different in A-MWCNT-treated mice. Data are the mean +/− SEM from 4 mice per group where 500 cells were counted from each animal randomly as described in *Methods*. **B**) Quantification of macrophages containing MWCNTs shows that approximately 50% of macrophages engulfed U-MWCNT or A-MWCNT after 1 day. 100 cells were counted in each BALF cytospin slides from U- or A-MWCNT-exposed mice. Data are mean +/− SEM of 4 animals in each group. Photomicrographs above each bar show representative inflammatory cells in BALF cytospin slides from U- or A-MWCNT-exposed mice where ‘Pos’ indicates MWCNT positive and ‘Neg’ indicates MWCNT-negative. Red arrows indicate neutrophils. **C**) Hematoxylin & eosin-stained lung sections at 1 day showing alveolar macrophages containing U-MWCNT or A-MWCNT (arrows). **D**) Representative microscopic images of trichrome-stained slides (20X) and semi-quantitative morphometry at 28 days following exposure to 0.1% pluronic/PBS vehicle (Control), U-MWCNT, or A-MWCNT.

Mice were exposed to a single dose of U-MWCNTs or A-MWCNTs (4 mg/kg) delivered by oropharyngeal aspiration (OPA). A-MWCNTs were coated with 50 ALD cycles of Al_2_O_3_ and the dose of A-MWCNTs corrected for estimated nanoparticle number relative to U-MWCNT. Differential counting was performed on BALF cells to determine relative percentages of macrophages, neutrophils, eosinophils and lymphocytes. As shown in **Fig. 6A**, exposure to either U-MWCNTs or A-MWCNTs caused significant increases in neutrophils and U-MWCNTs caused a slightly higher increase in eosinophils compared to A-MWCNTs. However, relative percentages of neutrophils or eosinophils were not significantly different between U- and A-MWCNT groups. Alveolar macrophages avidly engulfed either U-MWCNTs or A-MWCNT by 1 day post-exposure and approximately half of macrophages counted in BALF cytospin slides were found to contain visible inclusions of aggregated U- or A-MWCNTs in the cytoplasm (**Fig. 6B**). No significant differences in MWCNT-positive macrophages were observed between U- and A-MWCNT-exposed groups. Lung sections stained with hematoxylin and eosin (H & E) also showed MWCNT-containing macrophages in the alveolar region and around airways of the lower lung (**Fig. 6C**). While there were no significant differences between U- and A-MWCNT in the acute lung inflammatory response at 1 day, there were significant differences in lung fibrosis between these groups. U-MWCNT caused a significant increase in lung fibrosis by day 28 that was not seen with A-MWCNTs (**Fig. 6D**). Semi-quantitative scoring of lung sections showed U-MWCNTs caused a significant increase in the lung fibrosis score, whereas the fibrosis score for A-MWCNT treated mice was significantly lower.

### Al_2_O_3_ coating reduces levels of pro-inflammatory and pro-fibrogenic cytokines in the lungs of mice *in vivo*

**Figure 7:**
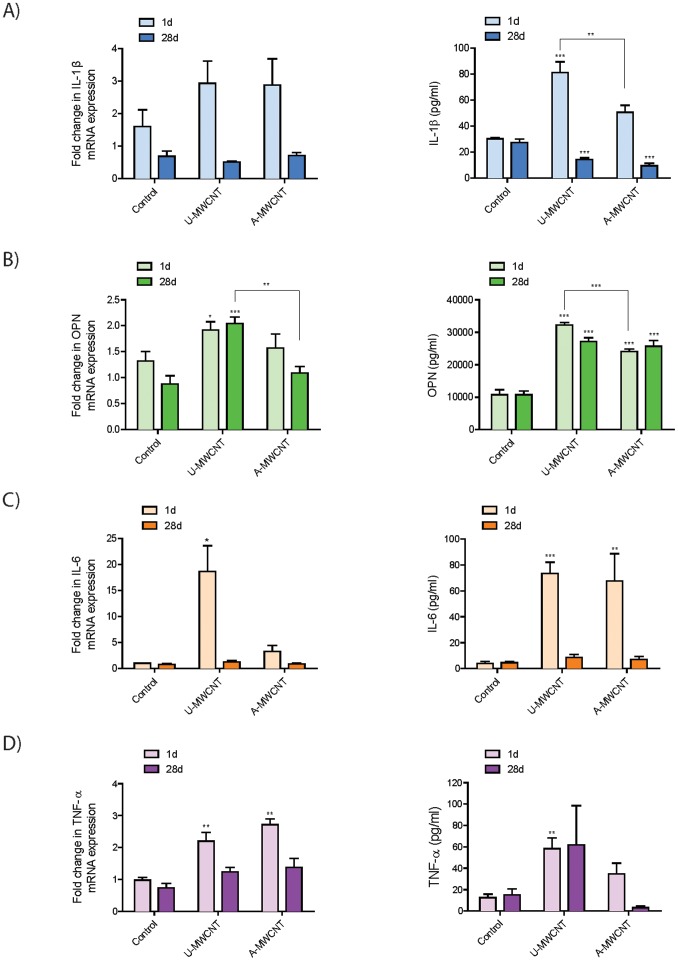
Effect of Al_2_O_3_-coated MWCNTs on cytokine protein levels in bronchoalveolar lavage fluid (BALF) in C57BL6 mice at 1 day or 28 days. Uncoated (U)-MWCNT or MWCNT coated with Al_2_O_3_ by 50 ALD cycles (A-MWCNT) were delivered to the lungs of mice by oropharyngeal aspiration (4 mg/kg) and necropsy performed at 1 or 28 days post-exposure to collect bronchoalveolar lavage fluid (BALF) and lung mRNA for measurement of **A)** IL-1β, **B)** OPN, **C)** IL-6 and **D)** TNF-α by ELISA and Taqman real-time RT-PCR, respectively.

In contrast to the increase in IL-1β caused by A-MWCNTs in human THP-1 and PBMCs *in vitro*, protein levels of IL-1β in mouse BALF were significantly decreased by A-MWCNTs as compared to U-MWCNTs (**Fig. 7A**). Levels of pro-IL-1β mRNA in lung were not different between A- and U-MWCNTs. Similar to the decrease in OPN caused by A-MWCNTs in THP-1 and PBMCs *in vitro*, protein levels of OPN in mouse BALF were significantly decreased at 1 day by A-MWCNTs relative to U-MWCNT (**Fig. 7B**). OPN protein levels were not different between U- and A-MWCNTs at 28 days. Lung OPN mRNA levels were also decreased by A-MWCNTs compared to U-MWCNTs at either 1 or 28 days. IL-6 protein levels in BALF were significantly induced by either U- or A-MWCNTs at 1 day but no significant differences were observed between U- and A-MWCNTs (**Fig. 7C**). However, lung mRNA levels of IL-6 were significantly increased by U-MWCNTs but not A-MWCNTs. TNF-α protein levels in BALF were significantly induced by U-MWCNTs but not by A-MWCNTs at 1 day (**Fig. 7D**). However, both U- and A-MWCNT increased lung TNF-α mRNA levels at day 1.

## Discussion

**Figure 8:**
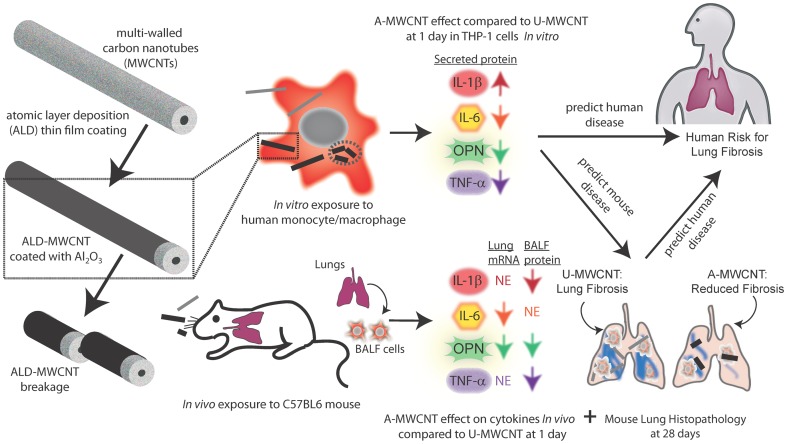
Illustration showing Al_2_O_3_ thin film coating of MWCNT by ALD and the effect on mononuclear cell cytokine production *in vitro* and pro-fibrogenic responses in the lungs of mice *in vivo*. Al_2_O_3_-coated MWCNTs (A-MWCNT) increased IL-1β secreted by THP-1 cells and PBMCs *in vitro*, but decreased IL-6, OPN, and TNF-α protein levels compared to uncoated (U)-MWCNTs. *In vivo*, ALD coating reduced lung mRNA levels of OPN and IL-6 but had no effect (NE) on lung mRNA levels of IL-1β or TNF-α at either 1 or 28 days post-exposure and reduced protein levels of IL-1β, OPN, and TNF-α measured in BALF at 1 or 28 days. ALD coating significantly reduced MWCNT-induced lung fibrosis in mice. Also, A-MWCNTs undergo breakage along the radial axis, which could potentially increase lung clearance of MWCNTs and reduce the potential for fibrosis.

An increasing number of studies demonstrate that multi-walled carbon nanotubes (MWCNTs) cause fibrosis when delivered to the lung of rodents, suggesting a human health risk. Moreover, a variety of post-synthesis modifications, including atomic layer deposition (ALD), are used to enhance the unique properties of MWCNTs. In this study we investigated whether aluminum oxide (Al_2_O_3_) surface coating applied to MWCNTs by ALD would alter the expression of pro-inflammatory or pro-fibrogenic cytokines *in vitro* using two human mononuclear cell models (THP-1 and PBMC) and whether ALD-coating would alter MWCNT-induced cytokine levels and fibrosis *in vivo* in the lungs of C57BL6 mice. MWCNTs coated with Al_2_O_3_ by ALD (A-MWCNT), when compared to uncoated (U)-MWCNTs, increased IL-1β secretion and yet decreased the production of IL-6, OPN, and TNF-α in cultured THP-1 cells and PBMCs *in vitro*. A-MWCNTs delivered to the lungs of mice by OPA had reduced mRNA or protein levels of IL-1β, IL-6, OPN and TNF-α relative to U-MWCNT at 1 or 28 days post-exposure and A-MWCNTs caused less lung fibrosis in mice relative to U-MWCNTs. These findings suggest that A-MWCNTs engineered by ALD with Al_2_O_3_ would reduce the risk of pulmonary fibrosis in humans compared to U-MWCNTs. A generalized scheme to depict the overall findings of this study is shown in **Fig. 8**.

The *in vitro* experiments with THP-1 cells and PBMCs showed that A-MWCNTs increased levels of IL-1β protein in cell supernatants but reduced IL-6, OPN, and TNF-α. Al_2_O_3_ nanoparticles also strongly induced IL-1β, but decreased IL-6, OPN, and TNF-α secretion in THP-1 cells. It is also possible that U- or A-MWCNTs, particularly at the high dose (100 mg/ml) that produced significant cytotoxicity, increased cytokine levels in cell supernatants by damaging cell membranes and releasing cytokines rather than actively inducing the secretion of the cytokines. The increased levels of IL-1β observed in THP-1 culture supernatants led us to predict that A-MWCNTs would have the potential to promote a greater acute lung inflammatory response but would reduce fibrosis in the lungs of mice as compared to U-MWCNTs. Consistent with this prediction, A-MWCNTs caused less lung fibrosis in mice at 28 days post-exposure compared to U-MWCNTs, but we did not observe any significant differences in the acute inflammatory response at 1 day post-exposure between A-MWCNT and U-MWCNT. Unlike the increase seen in IL-1β after A-MWCNT treatment in our cell culture studies, IL-1β protein in BALF in the lungs of mice was reduced as compared to U-MWCNTs and IL-1β mRNA levels were not different between A- and U-MWCNTs in mouse lung tissue. These data indicated that secretion of IL-1β by human mononuclear cells *in vitro* was not a reliable biomarker to predict IL-1β protein levels in the BALF from mice exposed to A- or U-MWCNTs. Inflammasome activation and subsequent secretion of IL-1β by macrophages are important components of the innate immune response to inhaled particles and fibers [28]–[32]. Previous reports demonstrate that MWCNTs cause inflammasome activation and subsequent IL-1β release by mononuclear cells to an extent similar to that of asbestos, a known cause of lung fibrosis [28]. It has been speculated that acute inflammation mediated by inflammasome activation and IL-1β release could be important to resolution and tissue repair after injury, whereas sustained inflammasome activation and IL-1β processing leads to fibrosis [31]. However, we observed that IL-1β levels in the BALF of mice were induced by U-MWCNT and to a lesser extent by A-MWCNT at 1 day post-exposure, and levels of IL-1β in either U- or A-MWCNT-treated mice returned to control levels by 28 days post-exposure. These findings suggest that soluble factors other than IL-1β account for differences in lung fibrosis in mice treated with U- or A-MWCNTs. Interestingly a recent study showed that MWCNTs stimulated acute neutrophilic inflammation in wild type mice but not in IL-1 receptor knock-out (IL-1R KO) mice [32]. However, the IL-1R KO mice develop more severe chronic fibrosis compared to wild type mice. Therefore, while IL-1β production is clearly linked to acute inflammation, its role in fibrosis is less clear and may depend on spatial or regional tissue expression.

Our data demonstrated that A-MWCNTs coated with Al_2_O_3_ suppressed OPN, IL-6, and TNF-α production by THP-1 cells and PBMCs compared to U-MWCNTs. Moreover, OPN protein was suppressed in the BALF of mice exposed A-MWCNTs compared to U-MWCNTs at 1 day post-exposure and TNF-α protein levels in BALF were suppressed at 1 and 28 days. OPN mRNA levels in lung tissue were reduced at 28 days post-exposure. OPN promotes the pathogenesis of pulmonary fibrosis by stimulating the migration, adhesion, and proliferation of lung fibroblasts [33]. Several lines of evidence indicate that OPN plays a central role in lung fibrosis. First, OPN is strongly up-regulated in the lungs of patients with idiopathic pulmonary fibrosis and expression appears to be independent of inflammation [34]. Second, OPN expression strongly correlates with fibrogenic lesion formation in the lungs of rodents exposed to bleomycin or nanoparticles, including titanium, cerium, and MWCNTs [34]-[38]. Third, work performed with OPN knock-out mice show that OPN mediates asbestos-associated lung injury and fibrogenesis [39]. Interestingly, serum OPN levels can be used to distinguish persons with exposure to asbestos who do not have cancer from those with exposure to asbestos who have pleural mesothelioma [40]. Therefore, OPN may be important in mediating potential carcinogenic effects of MWCNTs as well as fibrogenic effects. IL-6 and TNF-α also play important roles in the pathogenesis of pulmonary fibrosis. IL-6 serves a pro-fibrogenic role by promoting fibroblast cell survival via the activation of STAT-3 and activated STAT-3 opposes the pro-apoptotic effects of STAT-1 [21], [41]. TNF-α plays an important role in the pathogenesis of pulmonary fibrosis by increasing the production of TGF-β1, which in turn increases collagen synthesis by fibroblasts and differentiation of fibroblasts to myofibroblasts [42]. Therefore, IL-6 and TNF-α coordinately stimulate fibrogenesis by stimulating fibroblast survival and matrix deposition.

Monocytes or monocyte-derived macrophages were used in the present study for several reasons. First, macrophages are the first line of defense following toxic inhalation exposures and serve to clear inhaled particles or fibers from the lung [43]. PBMCs are precursors of lung macrophages and dendritic cells [44]. Second, MWCNTs delivered to the lungs of mice or rats are primarily found within macrophages within hours after exposure [14]–[16]. Finally, monocytes and macrophages produce a variety of soluble growth factors, cytokines, and extracellular matrix proteins that mediate tissue repair following injury or mediate the pathogenesis of fibroproliferative lung diseases, depending on macrophage phenotype and state of activation [21]. The human THP-1 macrophage cell line has been shown to produce increased levels of IL-1β after carbon nanotube exposure and thereby seemed to represent an appropriate cell type for determining the relative pro-inflammatory effects of ALD coated MWCNTs [27]. However, because THP-1 cells are a monocytic cell line derived from a patient with acute monocytic leukemia, it is possible that this cell line might not accurately reflect the responses of primary human monocyte/macrophages from healthy individuals. Therefore, we also evaluated primary PBMCs with or without LPS-priming. Our results obtained with primary PBMCs exposed to Al_2_O_3_-coated MWCNTs showed remarkable similarities to the results obtained with THP-1 cells; i.e., enhanced IL-1β release concomitant with markedly suppressed OPN production by Al_2_O_3_-coated MWCNTs or Al_2_O_3_ nanoparticles. However, we did not observe significant differences in IL-6 or TNF-α secretion between A- and U-MWCNT-treated PBMCs.

Aluminum exposure can cause toxic effects or therapeutic effects, depending on the chemical form of aluminum and the context of exposure. For example, Mice exposed to sub-chronic inhalation exposures of aluminum-oxide based nanowhiskers show increased numbers of lung macrophages, but no inflammatory or toxic responses [45]. Al_2_O_3_ nanoparticles are not overtly toxic to cultured human U937 macrophages *in vitro* but exposure impaired the cell's natural ability to respond to microbial pathogens [46]. Al_2_O_3_ nanoparticles have also been shown to act as an antigen carrier to reduce the amount of antigen required by dendritic cells to activate T cells in mice *in vivo* [47]. This study also showed that Al_2_O_3_ nanoparticles conjugated to tumor antigen resulted in tumor regression in mice. Thus, there is evidence that aluminum can either cause lung disease or can be engineered at the nanoscale to be relatively non-toxic or biocompatible. Chronic exposure to fumes containing aluminum has been shown to reduce lung function and cause pneumoconiosis in humans exposed in an occupational setting [48]. However, these fumes are likely a complex mixture and could contain agents other than aluminum. The effect of Al_2_O_3_-coated MWCNTs on chronic disease; lung fibrosis or cancer, remains to be determined and this will be an important focus of future work.

A variety of engineering methodologies have been developed to functionalize the surface of MWCNTs for the purposes of altering physical or chemical properties for specific applications, including amine modification, carboxylation, and ALD coating. Because functionalization is widely used to refine and enhance the properties of MWCNTs for a variety of applications, it is important to understand how altering the physical and chemical nature of MWCNTs will modify the toxicological response to these engineered nanomaterials. The ALD coating used in this study is increasingly used in nanotube modifications for variety of products and therefore represents a high probability for exposures. The potential risks of MWCNTs to human health, either unmodified or functionalized, are unknown. The current information available to predict the toxicity and biological effects of MWCNTs to humans is based on studies performed with cultured human or rodent cells and studies performed with rodents *in vivo*. The majority of these studies have been done with unmodified MWCNTs. Unmodified or ‘pristine’ MWCNTs cause inflammation and fibrosis in the lungs of mice or rats [14]–[19]. Other studies indicate that functionalization of MWCNTs alters the pathologic response. For example, carboxylated MWCNTs (COOH-MWCNTs) are more hydrophilic than pristine MWCNTs, and COOH-MWCNTs result in less prominent collagen deposition in the alveolar region in the lungs of mice [30].

While the Al_2_O_3_ coating on MWCNTs appears to be the major factor that alters cytokine production in THP-1 and PBMCs *in vitro*, nanotube length is still likely an important determinant of the inflammatory and fibroproliferative effects of MWCNTs in the lung *in vivo*. In general, long asbestos fibers or rigid MWCNTs (i.e., >20 µm) remain in the lung and are much more persistent than shorter fibers or nanotubes [20]. Therefore, the nanotube fragments resulting from breakage of A-MWCNTs coated with 50 or 100 ALD cycles of Al_2_O_3_ would likely be cleared from the lungs more rapidly than uncoated long MWCNTs or those coated with only 10 ALD cycles of Al_2_O_3_. We observed that the fracturing of A-MWCNTs occurred only after sonication prior to administration to cells *in vitro* or mice *in vivo*. However, unsonicated A-MWCNTs could be more likely to fracture over time in tissues as compared to U-MWCNTs. We did not address the issue of A-MWCNT clearance before or after fracturing in the present study, but future work should focus the relative clearance rates from the lungs of mice exposed to A-MWCNTs in comparison to U-MWCNTs. Another potentially important consideration is whether or not ALD coating with Al_2_O_3_ alters the formation of a protein corona around MWCNTs. It is possible that differences in cytokine levels in the supernatants from cells treated with U- or A-MWCNTs could be due to differences in protein corona formation around functionalized MWCNTs that could modify the adsorptive capacity of the nanomaterial. Characterization of the protein corona and the adsorptive capacity for cytokines after ALD modification of MWCNTs should be another important focus for future work.

The mass of individual MWCNTs was increased as a function of increasing cycles of ALD coating. In our initial experiments cells were dosed on a mass basis. This conventional strategy resulted in cells receiving fewer nanotubes since increasing ALD coatings progressively increased mass of the individual nanotubes. Accordingly, dosing cells on a mass basis resulted in a reduction of cellular responses (cytotoxicity, cytokine production) as a function of increasing ALD coatings. Therefore, based on the linear mass gain to ALD coating cycle relationship shown in **Fig. 1**, we were able to estimate numbers of nanotubes per unit mass for each series of ALD coating cycles to correct for the increase in mass and dose cells based on the estimated number of nanotubes.

In summary, we report that Al_2_O_3_-coated multi-walled carbon nanotubes (A-MWCNTs) functionalized by atomic layer deposition (ALD) altered cytokine production of human mononuclear cells *in vitro* and cytokine expression in the lungs of mice compared to uncoated MWCNTs (U-MWCNTs). A-MWCNTs also reduced lung fibrosis compared to U-MWCNTs. Reduced fibrosis was correlated with reduced mRNA or protein levels of IL-1β, IL-6, OPN, and TNF-α in the lungs of mice. Moreover, the reduced levels of IL-6, OPN, and TNF-α in mononuclear cells treated with A-MWCNTs *in vitro* predicted the reduced levels of these cytokines in the lungs of mice after A-MWCNT exposure. However, the elevated levels of IL-1β produced by mononuclear cells exposed to A-MWCNTs *in vitro* were not predictive of IL-1β levels that were reduced by A-MWCNT exposure *in vivo*. The combined *in vitro* and *in vivo* experimental approach presented here suggests that Al_2_O_3_-coated MWCNTs engineered by ALD would present a lower risk for human pulmonary fibrosis after inhalation exposure.

## Supporting Information

Figure S1 **Cell viability of THP-1 macrophages after exposure to aluminum oxide-coated MWCNTs for 24 hrs.** **A**) Cell viability data for THP-1 cells after being exposed to increasing doses of unsonicated and sonicated control (uncoated) and 10 ALD cycle Al_2_O_3_-coated MWCNTs. **B**) Cell viability data for THP-1 cells after being exposed to increasing doses of unsonicated and sonicated uncoated and 50 ALD cycle Al_2_O_3_-coated MWCNTs. **C**) Cell viability data for THP-1 cells after being exposed to increasing doses of unsonicated and sonicated uncoated and 100 ALD cycle Al_2_O_3_-coated MWCNTs. Graphs on the left represent a dose response in which dose was determined by the mass of the MWCNTs. Graphs on the right represent a dose response in which dose was based on nanoparticle number. Data are representative graphs of three separate experiments and are expressed as means ± SEM.
(TIFF)

Figure S2 **Cell viability of THP-1 cells after exposure to aluminum oxide and carbon black nanoparticles for 24 hrs.** MTS assay was performed on THP-1 cells after being exposed to increasing doses of sonicated carbon black vs. aluminum oxide nanoparticles.
(TIFF)

Figure S3 **Cell viability data for primary peripheral blood mononuclear cells (PBMCs) after being exposed to U-MWCNT, A-MWCNTs, CB and Al_2_O_3_ nanoparticles.** Cell viability measured by MTS assay as described in *Methods*. Data are expressed as the mean +/− SEM of quadruplicate cultures of PBMCs. *P<0.05, **P<0.01, ***P<0.001 compared to corresponding controls (−LPS or +LPS).
(TIFF)

Table S1 **Characteristics of nanotube suspensions used in the present study.** Dynamic Light Scattering (DLS) and Zeta potential analyses were performed as described in *Methods*.
(TIF)
