# Supplementary figures and images for: Atomic Layer Deposition Coating of Carbon Nanotubes with Aluminum Oxide Alters Pro-Fibrogenic Cytokine Expression by Human Mononuclear Phagocytes In Vitro and Reduces Lung Fibrosis in Mice In Vivo
Source: PLoS One. 2014 Sep 12;9(9):e106870. doi: 10.1371/journal.pone.0106870 (PMC4162563; doi:10.1371/journal.pone.0106870)

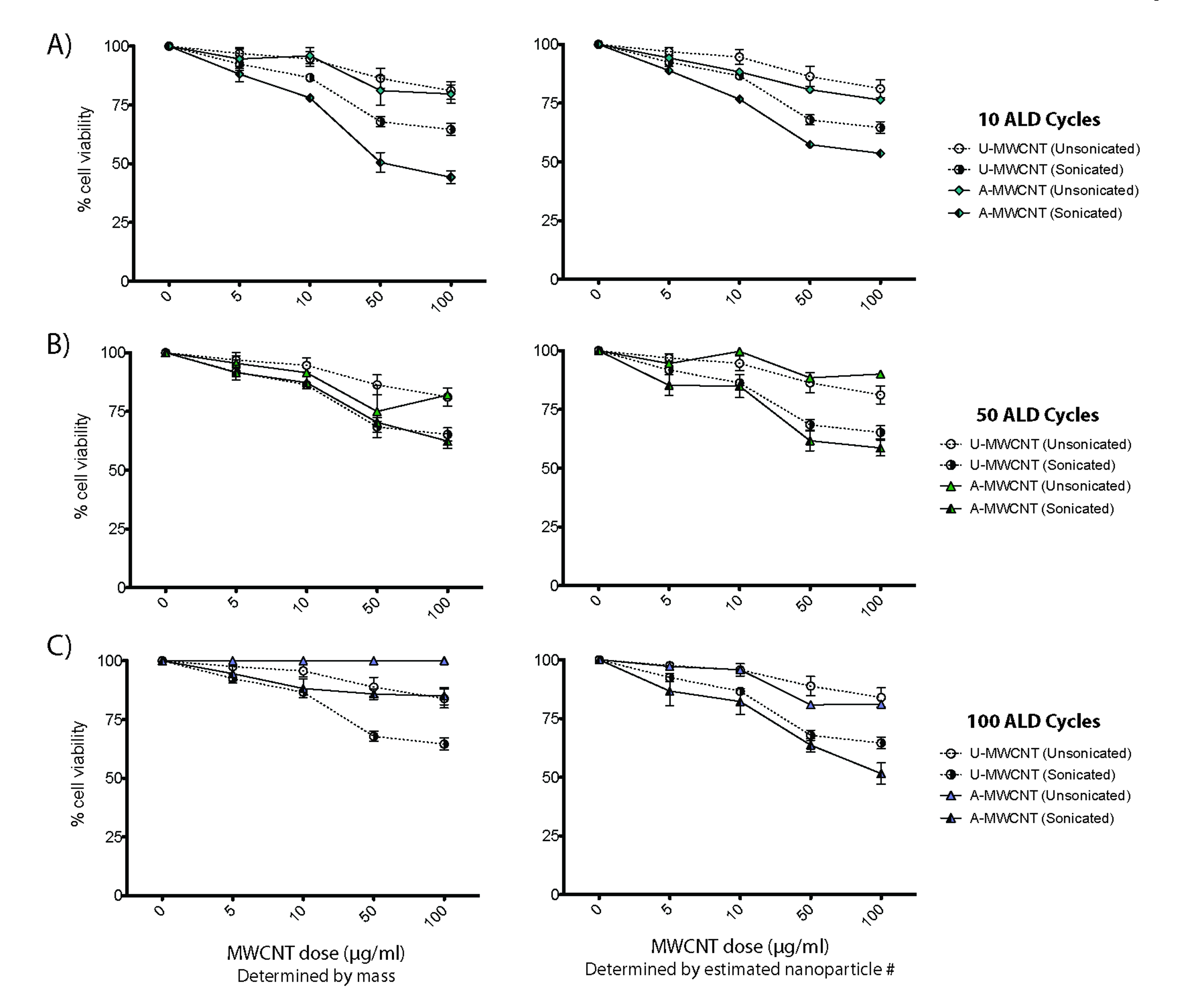

Supplement: Figure S1 — Cell viability of THP-1 macrophages after exposure to aluminum oxide-coated MWCNTs for 24 hrs. A) Cell viability data for THP-1 cells after being exposed to increasing doses of unsonicated and sonicated control (uncoated) and 10 ALD cycle Al2O3-coated MWCNTs. B) Cell viability data for THP-1 cells after being exposed to increasing doses of unsonicated and sonicated uncoated and 50 ALD cycle Al2O3-coated MWCNTs. C) Cell viability data for THP-1 cells after being exposed to increasing doses of unsonicated and sonicated uncoated and 100 ALD cycle Al2O3-coated MWCNTs. Graphs on the left represent a dose response in which dose was determined by the mass of the MWCNTs. Graphs on the right represent a dose response in which dose was based on nanoparticle number. Data are representative graphs of three separate experiments and are expressed as means ± SEM. (TIFF) [file pone.0106870.s001.tiff]

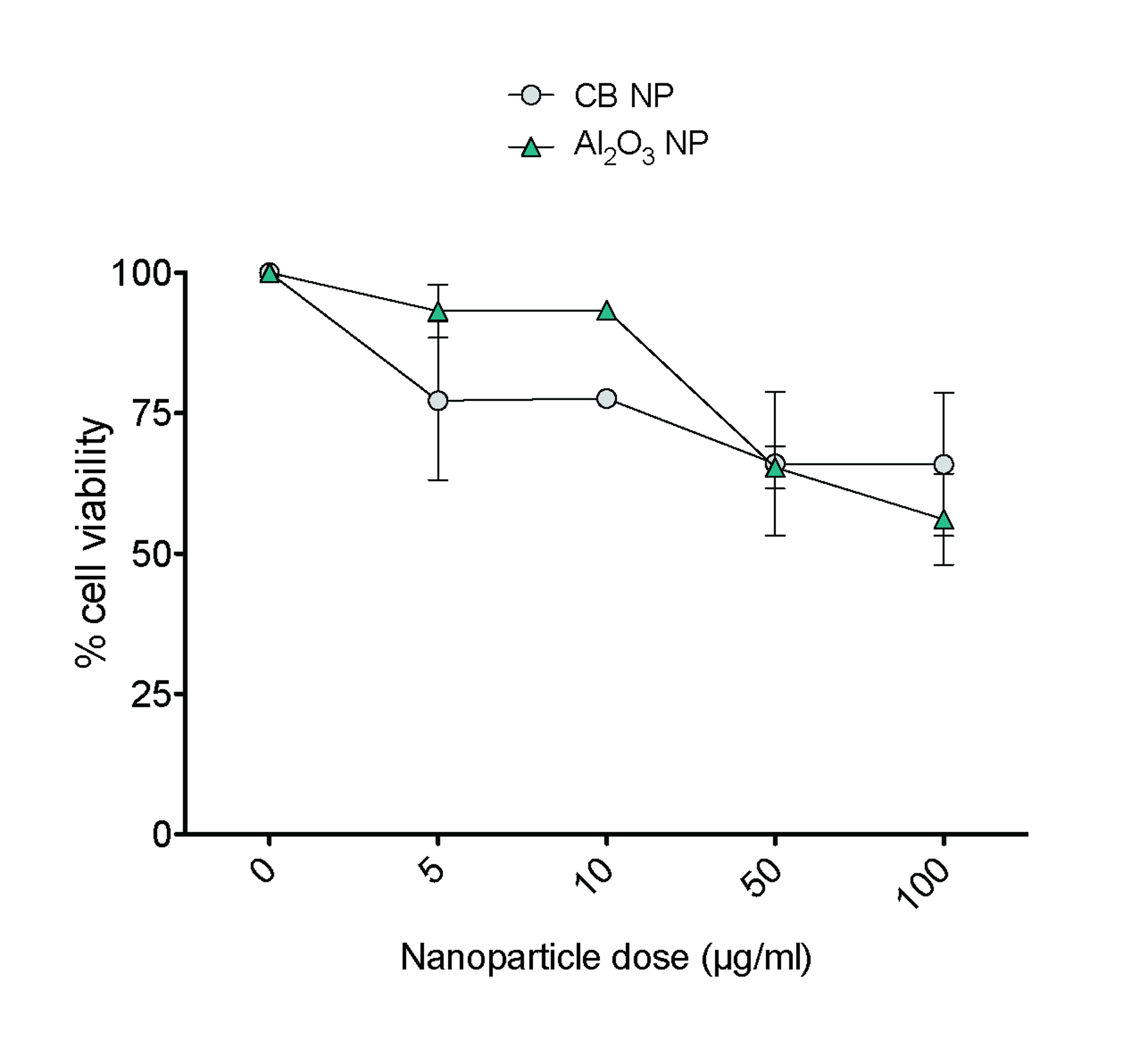

Supplement: Figure S2 — Cell viability of THP-1 cells after exposure to aluminum oxide and carbon black nanoparticles for 24 hrs. MTS assay was performed on THP-1 cells after being exposed to increasing doses of sonicated carbon black vs. aluminum oxide nanoparticles. (TIFF) [file pone.0106870.s002.tiff]

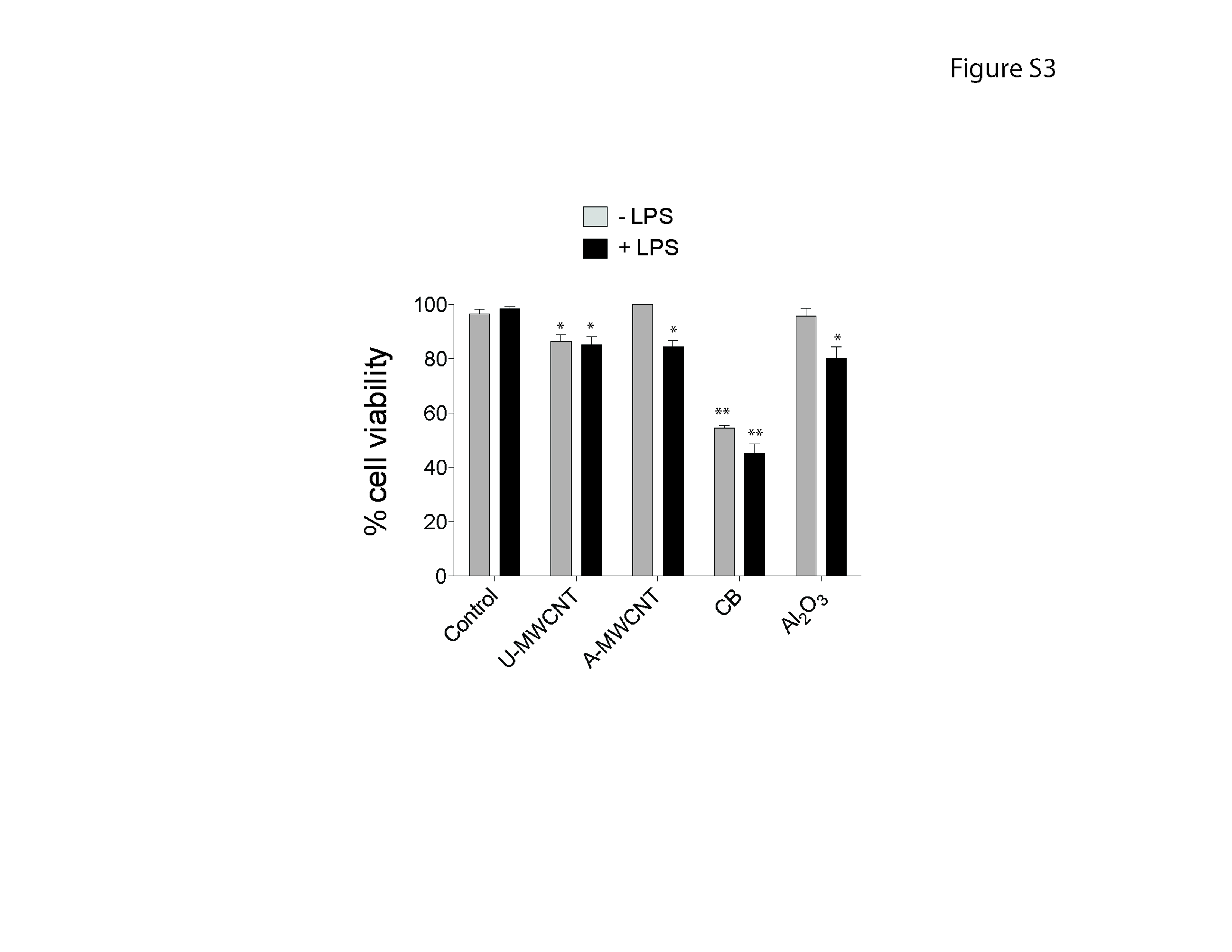

Supplement: Figure S3 — Cell viability data for primary peripheral blood mononuclear cells (PBMCs) after being exposed to U-MWCNT, A-MWCNTs, CB and Al2O3 nanoparticles. Cell viability measured by MTS assay as described in Methods . Data are expressed as the mean +/− SEM of quadruplicate cultures of PBMCs. *P<0.05, **P<0.01, ***P<0.001 compared to corresponding controls (−LPS or +LPS). (TIFF) [file pone.0106870.s003.tiff]

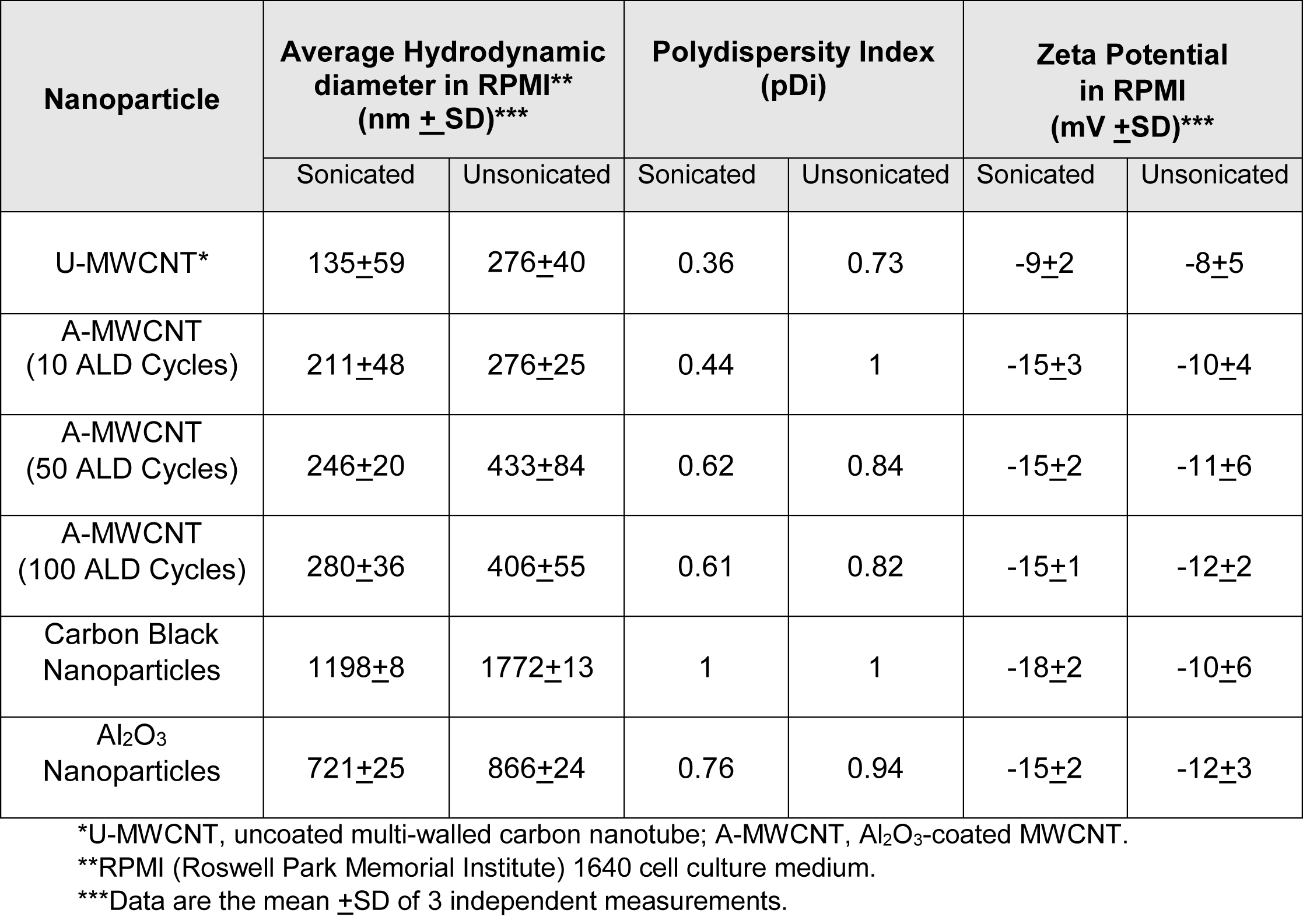

Supplement: Table S1 — Characteristics of nanotube suspensions used in the present study. Dynamic Light Scattering (DLS) and Zeta potential analyses were performed as described in Methods . (TIF) [file pone.0106870.s004.tif]
